# Phase variation in pneumococcal populations during carriage in the human nasopharynx

**DOI:** 10.1038/s41598-020-58684-2

**Published:** 2020-02-04

**Authors:** M. De Ste Croix, E. Mitsi, A. Morozov, S. Glenn, P. W. Andrew, D. M. Ferreira, M. R. Oggioni

**Affiliations:** 1 Department of Genetics and Genome Biology, University of Leicester, University Rd, Leicester, LE1 7RH United Kingdom; 2 Department of Clinical Sciences, Liverpool School of Tropical Medicine, Pembroke Pl, Liverpool L3 5QA United Kingdom; 3 Department of Mathematics, University of Leicester, University Rd, Leicester, LE1 7RH United Kingdom; 4 Department of Respiratory Sciences, University of Leicester, University Rd, Leicester, LE1 7RH United Kingdom

**Keywords:** Microbial genetics, Pathogens

## Abstract

*Streptococcus pneumoniae* is one of the world’s leading bacterial pathogens, responsible for pneumonia, septicaemia and meningitis. Asymptomatic colonisation of the nasopharynx is considered to be a prerequisite for these severe infections, however little is understood about the biological changes that permit the pneumococcus to switch from asymptomatic coloniser to invasive pathogen. A phase variable type I restriction-modification (R-M) system (SpnIII) has been linked to a change in capsule expression and to the ability to successfully colonise the murine nasopharynx. Using our laboratory data, we have developed a Markov change model that allows prediction of the expected level of phase variation within a population, and as a result measures when populations deviate from those expected at random. Using this model, we have analysed samples from the Experimental Human Pneumococcal Carriage (EHPC) project. Here we show, through mathematical modelling, that the patterns of dominant SpnIII alleles expressed in the human nasopharynx are significantly different than those predicted by stochastic switching alone. Our inter-disciplinary work demonstrates that the expression of alternative methylation patterns should be an important consideration in studies of pneumococcal colonisation.

## Introduction

*Streptococcus pneumoniae* is one of the major human pathogens, responsible for pneumonia, septicaemia and meningitis^1^. The primary niche of the pneumococcus is the human nasopharynx, where colonisation is often transient and asymptomatic^2,3^. In carriage studies conducted between 2001 and 2016 *S. pneumoniae* colonisation rate in UK children under 5 years old were estimated to be between 48–52% despite the introduction of vaccination programme^4^. Asymptomatic colonisation of the nasopharynx is considered a prerequisite for episodes of pneumonia and invasive infections such as septicaemia and meningitis. Pneumococcal diseases are the leading global cause of morbidity and mortality as a result of respiratory infection^1^. Despite this huge burden of disease, the mechanisms controlling the switch from harmless coloniser to invasive pathogen remain largely unknown.

There is a huge amount of genetic diversity in *S. pneumoniae*, largely resulting from its competence system, which allows the uptake and incorporation of exogenous DNA by homologous recombination^5,6^. Present in the genome of all isolates of *S. pneumoniae* is a type I restriction modification (R-M)system (SpnIII) which is capable of high frequency reversible recombination within the locus leading to different methylation states of the pneumococcal chromosome^6–9^. Phase-variable systems similar to SpnIII have been identified in a diverse range of bacterial species^10^ including *Mycoplasma pulmonis*^11^*, Bacteroides fragilis*^12^*, Listeria monocytogenes*^13^*, Enterococcus faecalis*^14^ and *Streptococcus suis*^15^.

The SpnIII system contains the classical type I R-M system genes of *hsdR (*restriction), *hsdM* (methylation) and *hsdS* (specificity), alongside two additional *hsdS* genes and a site specific recombinase, *creX*^6–8,16^. Only one of the *hsdS* genes found within the locus is a complete, functional gene and has been termed the active gene. The other two *hsdS* genes lack transcriptional start sites and act as donors of target recognition domains (TRDs). The presence of three inverted repeats between TRDs allows for shuffling between the active and silent genes. These variable *hsdS* genes result in the ability to methylate the bacterial chromosome at one of six different recognition sequences. The sequences recognised by these different TRD combinations are referred to as A-F^7,16^. These differential methylation patterns have previously been shown to impact the ability of *S. pneumoniae* to take up DNA following transfection and transformation^7,8,17,18^, but even more importantly to have epigenetic effects influencing the ability of SpnIII variants to colonise the murine nasopharynx and blood stream^7^ and on colony opacity variation^7–9^. However, no difference in the prevalence of active *spnIII hsdS* genes was found when comparing paired isolates from human cerebrospinal fluid or blood^19^.

In order to determine whether variation in the SpnIII methylation states also has the ability to impact on pneumococcal colonisation of the human and murine nasopharynx, we have analysed nasal wash samples from the Experimental Human Pneumococcal Carriage (EHPC) project^20^ and nasal lavages from 14 female CD1 mice colonised for 7 days^21^. In the EHPC model healthy volunteers were experimentally challenged with *S. pneumoniae* strain BHN418 and monitored for several weeks to determine levels of nasopharyngeal colonisation. For the murine colonisation model CD1 mice were colonised for 7 days using the same pneumococcal strain. The samples collected from these studies allowed for a longitudinal analysis of phase variation of the *spnIII* R-M system and investigation of the potential selection of alternative methylation patterns during colonisation of the human and murine nasopharynx^21^. This inter-disciplinary approach, using both mathematical modelling and analysis of human and mouse carriage samples, provides us with a unique opportunity to better understand the impact of stochastic genetic changes in pneumococcal carriage.

## Materials and Methods

### Growth and storage of bacterial strains

*S. pneumoniae* strains were grown on Brain Heart Infusion Agar (BHIA) supplemented with 3% v/v defibrinated Horse Blood (ThermoScientific, UK)^22–24^ For human intranasal inoculation, *S. pneumoniae* strain, serotype 6B (BHN418) was cultured until mid-log phase in Vegitone infusion broth (Fluka 41860, Sigma-Aldrich, Missouri, USA). To ensure analysis was only conducted on live bacteria, 10 µl of each nasal wash sample was spotted onto BHIA and incubated for 16–18 hours at 37 °C with 5% v/v CO_2_. All bacterial growth was collected and stored in 200 µl BHI with 10% v/v glycerol. All samples were stored at −80 °C. Pneumococcal serotype 6B strain BHN418^25^ carries an *spnIII* locus (Genbank ASHP01000014.1 and ASHP01000015.1) with three HsdS proteins with 99 to 100 percent amino acid identity with respective D39 orthologues^7^.

### Experimental human pneumococcal colonization (EHPC)

Bacterial samples were obtained from the experimental human pneumococcal challenge model wherein healthy volunteers are inoculated with live pneumococcus^20^. Briefly, healthy volunteers aged from 18–50, were inoculated intranasally with a well-characterized penicillin-sensitive 6B serotype pneumococcus (BHN 418). The inoculation dose was 80,000 CFU in 100 µl in saline per nostril. The strain was chosen because its genome is fully sequenced and there are negligible rates of natural colonization with 6B in Liverpool, UK. Nasal wash (NW) samples were collected pre-inoculation, and post-inoculation (Days 2, 7, 14, and 21). For pneumococcal detection, NW samples were centrifuged at 3,345 × g for 10 minutes^20^, followed by resuspension of the bacterial pellet in 100 μl of skimmed milk tryptone glucose glycerol medium. Serial dilutions of the pellet were plated on Columbia Horse Blood Agar containing 4 μg/ml gentamicin (Sigma, UK). Plates were incubated overnight at 37 °C with 5% v/v CO_2_ and inspected to identify pneumococcal phenotype. Serotype was confirmed by latex agglutination. Participants in whom the inoculated pneumococci were detected in NW samples at any visit post-inoculation were defined as experimentally colonized. All Colony Forming Unit (CFU) density data were calculated as CFU per ml of NW returned. All experimentally colonized participants who did not have two consecutive culture-negative NWs received amoxicillin, 500 mg three times per day, for 3 days at the end of the study to ensure 6B colonization clearance.

All volunteers gave written informed consent and research was conducted in accordance with all relevant ethical regulations. Ethical approval was given by the National Health Service Research Ethics Committee (REC). Ethics Committee reference number: 14/NW/1460.

### *hsdS* allele quantification

This analysis was conducted by plating of the nasal wash pellets of volunteers at all carriage-positive time points. Bacteria that grew on BHIA with 3% v/v horse blood were collected and analysed, those with <10 colonies were not analysed. No significant differences were found between samples analysed by DNA extraction from the NWP and those analysed by PCR amplification on cultured colonies (data not shown), this has previously been demonstrated by Manso *et al*.^7^ and Lees *et al*.^19^. Using nasal wash pellet samples^20^ collected at days 2, 7, 14 and 22–24, we were able to monitor changes in the active *spnIII* gene within a carriage episode. We also analysed the inoculum for each individual volunteer to allow for a direct comparison to the starting distribution and to enable a calculation of the rate of change over time.

Primers AMRE74L (5’FAM label) and AMRE59 were used to PCR amplify a 4.2 kb fragment as previously described^16^. Amplification was performed in a 25 µl reaction consisting of 0.75 µl 10 mM AMRE74L forward, 0.75 µl 10 mM AMRE59 reverse primer, 19.85 µl dH_2_O, 2.25 µl 11.1X buffer (for recipe see Jeffreys^26^), 0.2 µl Kapa Taq (5 U/µl) (Kapa Biosystems, UK), 0.15 µl Tris pH8.8, 0.05 µl PFU (2.5 U/µl) and 1 µl resuspended cells (as described in Growth and Storage of Bacterial strains) as template. All PCR reactions were performed as follows: denaturation at 95 °C for 5 min, followed by 40 cycles of 1 min denaturation at 95 °C, 1 min annealing at 68 °C, and 5 min extension at 68 °C, with a final extension of 10 min at 68 °C. 10–15 µl (depending on DNA concentration) of PCR product was digested following the manufacturer’s instructions using 1U DraI (New England Biolabs, UK), 2U PleI (New England Biolabs, UK), 1X CutSmart Buffer (New England Biolabs, UK) in a total volume of 20 µl. Following digestion each FAM labelled SpnIII fragment present in the amplification mix has a unique size that can be distinguished through capillary electrophoresis on an ABI prism Gene Analyser (Applied Biosystems, USA). Data received from the ABI Prism Gene Analyser were analysed using Peak Scanner v1.0 software.

### Mathematical modelling

The active SpnIII distribution in each of the inoculums received by volunteers was determined using the *hsdS* quantification method described. Once these data were obtained, it was possible to model the predicted stochastic changes to the active *Spn*III distribution within these populations in the absence of selection. Volunteers were grouped by statistically significant differences in the proportions of SpnIIIA in the inoculum resulting in three groups; group 1 (>50% A); group 2 (<30% A); and group 3 (>30 < 50% A). Only 5 volunteers fell into group 3 and this group was therefore excluded from further study as statistical analysis was not possible with such a small group size.

Temporal variation of the genetic composition of bacteria was modelled using the discrete Markov chain framework^27^. We make the following assumptions. At each time step (corresponding to cell division) recombination events only depend on the current state of the cell. We suggest that the recombination probabilities are time-independent and the growth rate of all genetic variants is the same. Possible recombination events in the model are determined by the so-called transition matrix. The structure of the transition matrix and its parametrisation can be found in the supplementary material. For simplicity, we assume a single inversion event per division. The elements of the transition matrix are actually recombination probabilities determined by a limited number of parameters which is much smaller (3 or 9) as the total size of the matrix (64). The elements of the transition matrix were estimated by fitting the experimental distributions of bacterial composition obtained when starting from different initial distributions of variants. Fitting model parameters to data was done via minimisation of the squares of deviations between the model and the data points. We assumed active replication of the pneumococcus within the nasopharynx during episodes of colonisation, therefore all modelling was conducted assuming 12 generations per day for 22 days (264 generations), unless otherwise stated.

### Murine colonisation model

A total of 14 female CD1 mice (bred in house at the University of Leicester) were intranasally colonised with 5 × 10^4^ CFU of *S. pneumoniae* BHN418 suspended in 10 µl BHI^28–30^. Mice were anaesthetised using 2.5% (v/v) isoflorane at 1.6–1.8 litre O_2_/min in an anaesthetic box. The challenge dose was delivered by inhalation while mice were held in a horizontal position. 7 days after inoculation, blood was collected under terminal anaesthesia and mice were culled by a schedule 1 method. After confirmation of death, lung and nasal washes were collected using 500 µl of BHI with 10% v/v glycerol, washes were repeated 3 times^31^. This work was conducted under Project Licence 60/4327. All procedures were approved by the University of Leicester Animal Welfare and Ethical Review Body (AWERB) and were in accordance with Home Office licence P7B01C07A.

## Results

**Figure 1:**
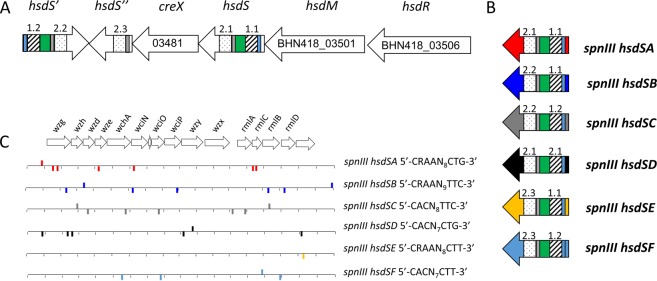
Arrangement of the *S. pneumoniae spnIII* locus. In strain BHN418 the *spnIII* locus **(A)** is split across two contigs with part of *hsdS* (BHN418_03476), *creX* (BHN418_03481), *hsdS*’ (BHN418_03486) and *hsdS*” (BHN418_03491) on contig 14 (GenBank ASHP01000014) and part of *hsdS* (BHN418_03496), *hsdM* (BHN418_03501), and *hsdR* (BHN418_03506) on contig 15 (GenBank ASHP01000015). This sequence has 99% protein identity to the same sequence in *S. pneumoniae* D39 (Manso^7^). In the orientation shown the active *hsdS* gene of the *spnIII* locus is an SpnIIIA (TRD1.1, 2.1), this results in the genome being methylated at sites recognised only by SpnIIIA. Recombination on inverted repeats within the locus will shuffle TRDs and can generate five other possible active *hsdS* genes **(B)**. Hashed boxes represent TRD 1 positions (TRD1.1 and 1.2), dotted boxes represent TRD 2 positions (TRD 2.1, 2.2 and 2.3). Blue boxes represent an 85 bp inverted repeat, green boxes a 330 bp inverted repeat and grey boxes a 15 bp inverted repeat. **(C)** Shown in panel C are the methylation sites in the capsule locus of strain BHN418 (GenBank ASHP01000006), sites were mapped using known methylation patterns in *S. pneumoniae* D39^7,8^. *hsdS* genes **(B)** and corresponding methylation sites **(C)** are shown in matching colours as follows: *hsdSA –* red, *hsdSB –* dark blue, *hsdSC –* grey, *hsdSD* – black, *hsdSE* – yellow, *hsdSF –* light blue.

The *spnIII* locus of *S. pneumoniae* is capable of a series of genetic rearrangements that result in the shuffling of TRDs between active and silent genes. The *spnIII* locus of BHN418 has an identical organisation to the DNA locus with 99–100% amino acid sequence identity between equivalent coding regions (Fig. 1A). The alternative combinations of TRDs allow six different *hsdS* genes to be generated (Fig. 1B), resulting in changes to the m-6A methylation of the whole pneumococcal genome, including the capsule locus (Fig. 1C)^7^. Previous work has shown that these alternative methylation patterns can lead to significant differences in the nasopharyngeal colonisation of mice^7^.

To establish if this also occurs in episodes of human carriage of pneumococci, we utilised samples from a human volunteer study wherein individuals were challenged with the BHN418 pneumococcal strain. Samples from 41 carriage-positive volunteers were analysed to determine the distribution of active *hsdS* genes (Fig. 1B) in *S. pneumoniae* populations over time.

**Figure 2:**
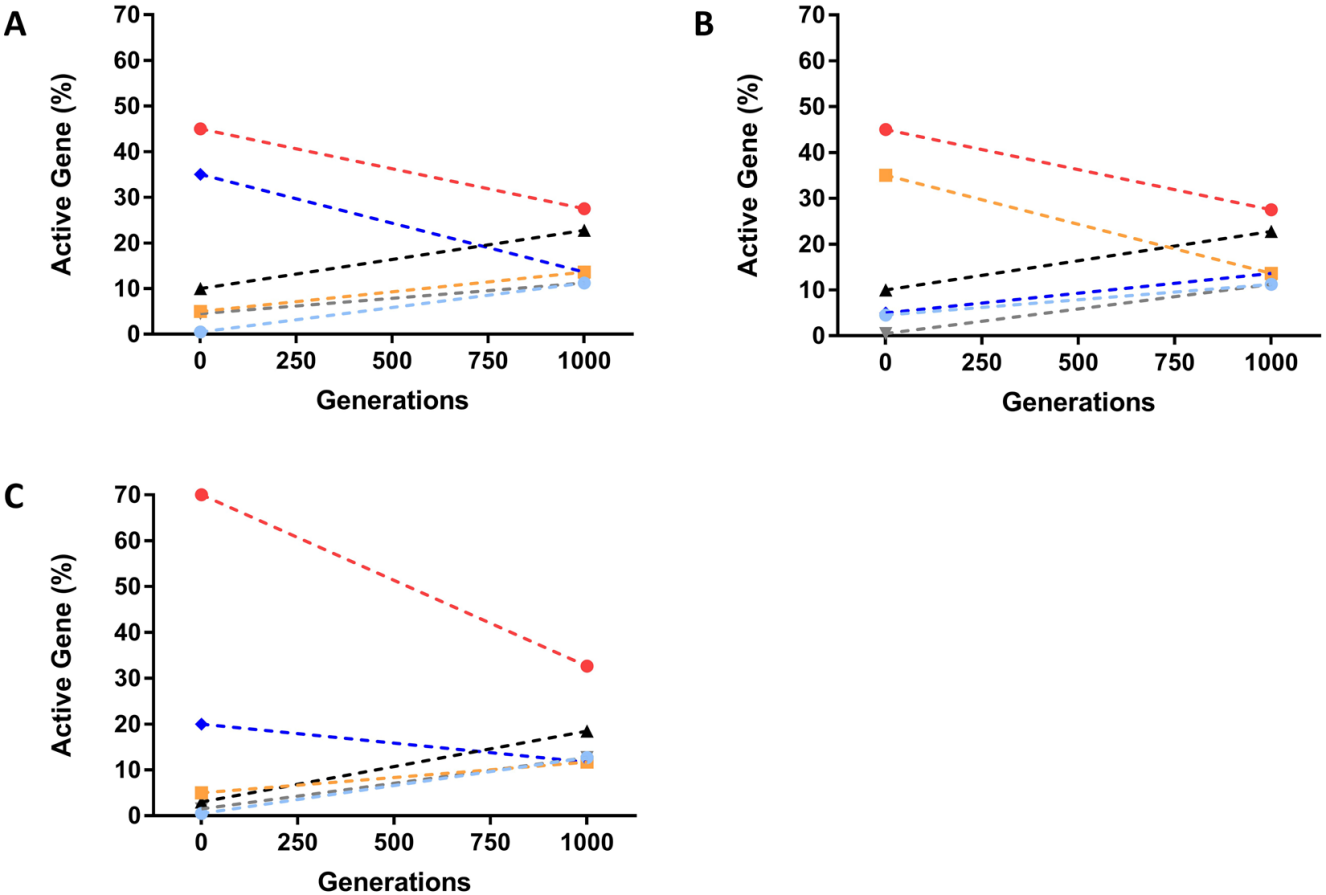
The predicted change in expression of active *hsdS* genes over 1000 generations. If all TRD switching between *hsdS* genes is modelled stochastically and not influenced by selection we observe a move towards equilibrium after 1000 generations (**A** and **B**). However the model demonstrates that the initial orientation of the locus does impact the relative abundance of each active gene within the population until this equilibrium is achieved. A population with 45% A1 (**A**) does not show the same distribution of active genes at 250 generations as a population with 45% A2 (**B**). In the absence of selection all populations will meet an end equilibrium irrespective of the initial starting population, although populations dominated by a single active gene will require more generations to reach equilibrium (**C**). Shown are the modelled outcomes of different mixed *hsdS* populations with *hsdSA* (red), *hsdSB* (dark blue), *hsdSC* (grey), *hsdSD* (black), *hsdSE* (yellow) and *hsdSF (*light blue) represented by dashed lines.

The structure of the SpnIII locus facilitates recombination between the different TRDs and as a result the composition of the starting population can have a significant impact on the output population (Fig. 2). Populations with a skew in one direction (Fig. 2C) will take more generations to reach the same end point as a population that is initially more evenly distributed (Fig. 2A,B). Due to the structure of the locus there are two potential orientations of the locus when *hsdSA* and *hsdSD* are active. These two orientations have been termed A1/A2, and D1/D2. These additional orientations could not be detected by the *hsdS* quantification method used within this study, however they can be calculated from the rate of switching between *hsdSB* and *hsdSE* (for A1/A2) and *hsdSC* and *hsdSF (*for D1/D2).

**Figure 3:**
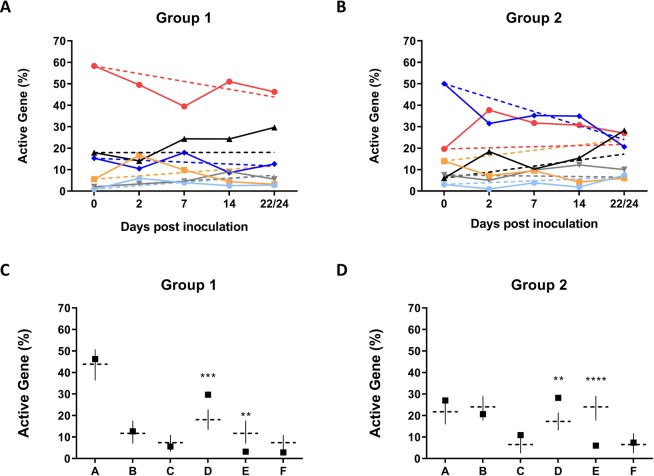
The observed and modelled active *hsdS* distribution in pneumococci in the human nasopharynx. The initial active *hsdS* gene distributions within inoculating doses of *S. pneumoniae* BHN418 were experimentally determined. This represents time point zero for both modelled (dashed lines) and experimentally quantified (solid lines) datasets (panels A and B). Experimentally quantified time points include all volunteers carriage positive by PCR. Shown in panels A and B are the experimental (solid line) and modelled (dashed line) outcomes of *S. pneumoniae* populations in the human nasopharynx are shown as *hsdSA* (red), *hsdSB* (dark blue), *hsdSC* (grey), *hsdSD* (black), *hsdSE* (yellow) and *hsdSF (*light blue). When the experimental outcomes of group one (panel C) and group two (panel D) were compared to the outcome predicted by the model there was a significant over representation of *hsdSD* at 22–24 days (p < 0.001 (**C**), P = 0.01(**D**)), and a significant under representation of *hsdSE* (p = 0.01 (**C**), p < 0.001 (**D**)). Model range is shown by the vertical line, mean percentage of active genes is shown by squares. The mean percentages of *hsdSD* and *hsdSE* are the only active genes to fall outside the range predicted by the model. Significance was tested used a student’s T test with a Holm-Sidak correction, *p < 0.05, **p = 0.01, ***p = 0.001, ****p < 0.001.

In order to determine whether the differences observed in the EHPC samples were due to stochastic switching or a continuing selection mechanism within the human nasopharynx, the experimentally observed distributions were compared to those predicted by a mathematical model. The experimental data showed a high level of variability both between and within individual volunteers at all time points, with an overall trend towards an equilibrium between the six active *hsdS* genes (Fig. 3A,B). The *hsdS* gene distributions for carriers positive at three weeks post inoculation were compared to outputs from the model.

For four of the six active genes there were no significant differences between the experimentally detected percentage and that predicted by the model (Fig. 3C,D). However, for *hsdSD* and *hsdSE* there was a significant over (*hsdSD*, p < 0.001) or under representation (*hsdSE*, p < 0.001) in the experimental samples, when compared to that predicted by the model. This outcome was found in both group 1 (Fig. 3C) and group 2 (Fig. 3D) where the starting proportions for *hsdSD* and *hsdSE* were 17.9% and 5.5% and 6.0% and 14.0% respectively. The model predicts that over time, in the absence of selection, *hsdSD* would represent 25% of the overall population, whereas in group 1 (Fig. 3C) *hsdSD* contributes a mean 29.6% of all populations analysed while in group 2 the mean was 28.2%. The higher values for the experimental results suggest that a weak selective pressure maintains the proportion of the population actively expressing *hsdSD*. Analysis of sample distribution at the earlier time points (days 2, 7 and 14) show a high level of variability between individuals at the same time point, as well as within the same individual at different time points (Fig. S1), suggesting recurring bottlenecks within the nasopharynx, which may be unrelatedly to any selective pressure.

**Figure 4:**
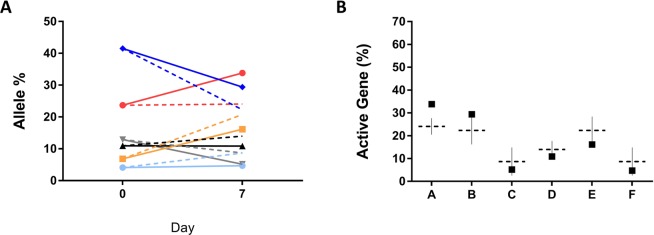
The observed and modelled active *hsdS* distribution in the murine nasopharynx. All data is pooled from 12 mice nasally challenged with a low dose of *S. pneumoniae* BHN418 that showed no signs of disease and had no detectable bacterial load in their blood. The initial active *hsdS* gene distributions within the inoculating dose of *S. pneumoniae* were experimentally determined. This represents time point zero for both modelled (dashed lines) and experimentally quantified (solid lines) datasets (panel A). The experimental and modelled outcomes of *S. pneumoniae* populations in the murine nasopharynx are shown as *hsdSA* (red), *hsdSB* (dark blue), *hsdSC* (grey), *hsdSD* (black), *hsdSE* (yellow) and *hsdSF (*light blue). When the experimental outcome was compared to the outcome predicted by the model (Panel B) no significant differences were observed. Significance was tested used a student’s T test with a Holm-Sidak correction, *p < 0.05, **p = 0.01, ***p = 0.001, ****p < 0.001.

In order to further examine changes in the *hsdS* proportion during carriage we also undertook a carriage experiment in mice. CD1 mice were challenged with *S. pneumoniae* BHN418 and monitored for 7 days. At the dose used, mice showed no signs of disease and had no detectable bacterial load in the blood. Both the inoculum and extracted pneumococcal populations were examined for their *hsdS* gene profiles. The expected outcome of the inoculum was examined in our mathematical model and compared to the experimentally observed pooled data from all 14 mice (Fig. 4). No significant differences in any active *hsdS* gene were observed. Thus the murine colonisation model did not reproduce the differences observed after three weeks of human carriage. As a result, we performed a further analysis of the human dataset and found that there were no significant differences in any *hsdS* gene at seven days post colonisation (Fig. S2).

## Discussion

The type I R-M System SpnIII (Fig. 1) is a highly conserved set of genes capable of undergoing frequent, stochastic genetic rearrangements that lead to different genomic methylation patterns in *S. pneumoniae*. These changing methylation patterns have been shown to influence factors such as capsule production^7–9^ and survival within murine models of infection^7^. However, no previous work has studied these genetic rearrangements in the natural niche of the pneumococcus, the human nasopharynx.

An analysis of *S. pneumoniae* populations recovered from human meningitis cases^19^ did not find evidence of selection for any of the six *hsdS* genes in either the blood or cerebral spinal fluid (CSF) samples. In this case the lack of selection was determined as no individual SpnIII variant showed a recurring dominance across more than 600 bacterial isolates, belonging to multiple serotypes and sequence types. As the input population will always be the unknown parameter for clinically recovered *S. pneumoniae* strains, a meaningful analysis to determine population selection is challenging. Importantly, samples from the EHPC^20^ with a known input population, and multiple sampling points, provided an ideal model to monitor any potential selection of *hsdS* genes during the course of human carriage. Unlike the work of Lees *et al*.^19^ we have examined pneumococcal carriage populations of a single pneumococcal strain not those obtained from cases of disease caused by many different serotypes and sequence types. As recently shown, SpnIII variant distribution can show huge variability across different sequence types and serotypes^16^, as a result evidence of selection may have been masked in the previous study.

Previous work has found that invasive pneumococcal infections, such as bacteraemia, and pneumococcal transmission can result in single cell bottlenecks^22,32^. The finding of Gerlini *et al*., that mice infected with multiple isogenic variants of *S. pneumoniae* develop bacteraemia caused by a single isogenic variant, strongly suggests that bottlenecks are an important feature in the development of invasive pneumococcal disease. The data from our model (Fig. 2) suggests that if bottlenecks are also occurring in the nasopharynx this may distort the observed population outcomes. The impact of population bottlenecks, such as those reported by Gerlini *et al*.^22^, on the selection of different phase variable profiles (known as phasotypes) has not previously been investigated in the pneumococcus. There is however data showing that the generation of bottlenecks in the intestinal tract has a significant impact on the phasotype of *Campylobacter jejuni*^33^. In this work chickens were inoculated with a mixed dose of phase variants but populations recovered from individual chickens were dominated by a single phasotype resulting from a presumed single cell bottleneck during the establishment of colonisation^33^. We considered whether bottlenecks in the nasopharynx may explain our observed results. The high levels of variability observed at the *spnIII* locus during human carriage episodes suggests that there may be continuous, on-going bottlenecks (Fig. S1) during prolonged carriage in each individual. The re-generation of a population from a small number of cells would create populations randomly skewed towards a single *spnIII* type as observed for SSR variants in *C. jejuni* by Wanford *et al*.^33^. In the absence of selection, starting distribution and switching rate will impact the chance of a particular variant founding the next dominant epigenetic profile of a population, however the distribution is likely to be relatively equal. Our observation that one active *hsdS* gene (*hsdSD)* is over-represented, suggests that the process is not random, as this variant is not over-represented in the starting populations. It could however also indicate an absence of selective bottlenecks, when considered individually (Fig. S1) there is evidence for bottlenecks within individual volunteers, however it is not possible to determine if these are random or the result of selective pressures.

The proposed over- or under- representation of active *hsdS* genes within the human nasopharynx is indicative of epigenetic selection where certain epigenetic states are favoured by a specific environment or set of selective pressures. The over-representation of *hsdSD* suggests that this phenotype is better suited to adherence in the nasal passages or avoids detection by the immune system at the mucosal membranes. However, the results of the human carriage study were not replicated in our study in mice, a result which may be explained by a variety of factors. Murine colonisation was assessed at 7 days, this potentially limits the time available for a single active *hsdS* gene to gain an advantage after an initial population bottleneck or does not allow enough time for selection to drive the population into the *spnIIID* state. Alternatively, it may be that the cells expressing an active *hsdSD* only have an advantage once the population has been established, while the maintenance of an increased level of diversity could be beneficial in the initial stages of colonisation. In addition, differences in the ligands for adhesins or immune defense molecules may have prevented coalescence to the same epigenetic state. Finally it must be considered that the mouse is not the natural host of the pneumococcus, therefore the expression of and methylation by *hsdSD* may lead to gene expression differences^7^ that are beneficial within human tissues but offer little or no advantage in a mouse, or at least within a CD1 mouse. Further testing of other mouse genotypes would be required to determine if this result can be applied to alternative mouse models.

An additional consideration is that the human nasopharynx is a source of phages capable of infecting *S. pneumoniae* as recently demonstrated by Furi *et al*.^18^. This work also shows that the number of SpnIII sites present in a phage genome will determine how likely it is to be successfully restricted by the infected cell, and therefore determine the likelihood of that cell’s survival. The variation in phages present in different individuals, as well as the number of SpnIII sites within those phage genomes, could have a significant impact on the population level expression of different SpnIII specificities. Importantly these phages will be absent from the murine nasopharynx offering further explanation for the differences observed between the human and murine models.

The work presented here investigated *hsdS* expression during pneumococcal carriage, however the study of *hsdS* selection within invasive and non-invasive infections has previously demonstrated that *S. pneumoniae* D39 cells expressing alternative *Spn*III methylation patterns show differing abilities to colonise the nasopharynx or survive in the bloodstream of mice^7^. These differences in the survival of SpnIII variants in different host niches was determined in experimental colonisation and sepsis models in CD1 mice, while the present work has utilised both human and mouse studies. In addition, previous published work on SpnIII has used phase-locked mutants^7–9^, incapable of expressing alternative SpnIII variants. While valuable for determining the impact of a specific methylation state it does not allow for an investigation into the dynamics of phase variation at the locus. The work presented here offers an insight into the on-going, reversible changes in this common inhabitant of the human nasopharynx.

The ability to subtly change global gene regulation patterns through the modification of methylation patterns offers a potential explanation for the poorly understood switch between harmless pneumococcal colonisation and a potentially fatal invasive infection. Alternatively SpnIII variability and variation in this locus is a defense against attack by bacteriophages naturally resident within the oral cavity and nasopharynx.

## Supplementary information

Supplementary Dataset 1.

## Data Availability

All data generated or analysed during this study are included in this published article (and its supplementary information files).
