# Author Correction: Phase variation in pneumococcal populations during carriage in the human nasopharynx

**DOI:** 10.1038/s41598-020-66187-3

**Published:** 2020-06-08

**Authors:** M. De Ste Croix, E. Mitsi, A. Morozov, S. Glenn, P. W. Andrew, D. M. Ferreira, M. R. Oggioni

**Affiliations:** 1 Department of Genetics and Genome Biology, University of Leicester, University Rd, Leicester, LE1 7RH United Kingdom; 2 Department of Clinical Sciences, Liverpool School of Tropical Medicine, Pembroke Pl, Liverpool, L3 5QA United Kingdom; 3 Department of Mathematics, University of Leicester, University Rd, Leicester, LE1 7RH United Kingdom; 4 Institute of Ecology and Evolution, Russian Academy of Sciences, 33 Leninskii pr., Moscow, 119071 Russia; 5 Department of Respiratory Sciences, University of Leicester, University Rd, Leicester, LE1 7RH United Kingdom

Correction to: *Scientific Reports* 10.1038/s41598-020-58684-2, published online 04 February 2020

The original version of this Article omitted an affiliation for A. Morozov. The correct affiliations for A. Morozov are listed below:

Department of Mathematics, University of Leicester, University Rd, Leicester, LE1 7RH, United Kingdom.

Institute of Ecology and Evolution, Russian Academy of Sciences, 33 Leninskii pr., Moscow 119071, Russia

This has now been corrected in the HTML and PDF versions of this Article, and in the accompanying Supplementary Material.

