# Supplementary material for: Phase variation in pneumococcal populations during carriage in the human nasopharynx
Source: Sci Rep. 2020 Feb 4;10:1803. doi: 10.1038/s41598-020-58684-2 (PMC7000782; doi:10.1038/s41598-020-58684-2)
Supplement: Supplementary file 1 — Supplementary Dataset 1. [file 41598_2020_58684_MOESM1_ESM.pdf]

Phase variation in pneumococcal populations during carriage in the human nasopharynx

Supplementary Materials

M. De Ste Croix[1], E. Mitsi[2], A. Morozov[3], S. Glenn[4], P.W. Andrew[4], D. M. Ferreira[2] and M. R. Oggioni[1]*

Supplementary materials

A Markov chain model was developed to mimic the temporal variation of genetic composition of bacteria[1]. The main assumptions of the model are the following: (i) at each time step (cell division) mutations only depend on the current state of the cell (this is known as the Markov property), (ii) mutation probabilities are time-independent, and (iii) the growth (replication) rate of all genetic variants is assumed to be the same. The elements of the transition matrix determine the recombination probabilities at each division. To mimic possible mutations in the considered system the transition matrix should possess a particular structure (e.g. a large number of elements will be zeros); the detailed description of the matrix is provided below. The non-zero elements depend on a small number of parameters which were estimated by fitting experimental distributions of bacterial composition obtained when starting from different initial distributions of variants. The corresponding pseudo-code required to fit model parameters to empirical data is discussed in detail below.

Model equations and parameters fit

Dynamics of variation of genetic composition of bacteria was modelled using the following discrete Markov chain with a stationary matrix[1]

$$X_{n+1} = X_n * P = X_0 * P^n,$$

where X_n is a row vector describing the percentage of different genetic variants within the population after n divisions (n =0 corresponds to the initial distribution of variants); the sum of all elements of X_n should always add up to 1; P is the transition matrix of size 8x8 modelling inversion rates, i.e. transition between different variants within a single cell division. The matrix P is a Markov (stochastic) matrix, i.e. the sum of the elements of each row is always equal to 1, i.e. $\sum_{j=1}^{8} p_{i,j}=1$. The meaning of each element $p_{i,j}$ is the probability that the variant 'i' will invert (recombine) and become 'j'.

In the vector X_n, we consider the following order of variants: (A_1, A_2, B, C, D_1, D_2, E, F).

The transition matrix P has the form described by the table below.

	A_1	A_2	B	C	D_1	D_2	E	F

**A_1**	$1-\alpha_1-\beta_1-\gamma_1$	β_1	α_1	γ_1	0	0	0	0
**A_2**	β_1	$1-\alpha_1-\beta_1-\gamma_1$	0	0	0	0	α_1	γ_1
**B**	α_2	0	$1-\alpha_2-\beta_2-\gamma_2$	0	γ_2	0	β_2	0
**C**	γ_2	0	0	$1-\alpha_2-\beta_2-\gamma_2$	α_2	0	0	β_2
**D_1**	0	0	γ_1	α_1	$1-\alpha_1-\beta_1-\gamma_1$	β_1	0	0
**D_2**	0	0	0	0	β_1	$1-\alpha_1-\beta_1-\gamma_1$	γ_1	α_1
**E**	0	α_3	β_3	0	0	γ_3	$1-\alpha_3-\beta_3-\gamma_3$	0
**F**	0	γ_3	0	β_3	0	α_3	0	$1-\alpha_3-\beta_3-\gamma_3$

Here the coefficients α_i, β_i, γ_i (i=1,2,3) give the probability of the corresponding recombination which occurs via a single inversion event. Initially, we neglected the probability of having more than one inversion event at a time.

The elements $p_{i,j}$ in P were found by fitting the model to experimental data which provided the eventual distributions of variants starting from different initial conditions consisting of pure variants A_2, B, C, D_2, E and F. We assume that the final experimental data sets approximately corresponded to n=20 cell divisions. As a formal criterion of goodness of fit to define the parameters α_i, β_i, γ_i, we minimised the following score Q which is the sum of squares of the norms of deviations between the model prediction and the data

$$Q(\alpha_i, \beta_i, \gamma_i) = \sum_{i=1}^{k_A} \|X_{20}(A_2) - Y_i(A_2)\|^2 + \sum_{i=1}^{k_B} \|X_{20}(B) - Y_i(B)\|^2 + \sum_{i=1}^{k_C} \|X_{20}(C) - Y_i(C)\|^2 + \sum_{i=1}^{k_D} \|X_{20}(D_2) - Y_i(D_2)\|^2 + \sum_{i=1}^{k_E} \|X_{20}(E) - Y_i(E)\|^2 + \sum_{i=1}^{k_F} \|X_{20}(F) - Y_i(F)\|^2,$$

where $X_{20}(Z)$ describes the distribution of variants in the model after n=20 divisions starting from 100% of the variant Z; $Y_m(Z)$ denotes the empirical distribution in experiment m (where m=1,…, k_Z) starting from 100% of the variant Z, where Z is one of A_2, B, C, D_2, E, F. Here, by the norm $\|X\|$ of a vector X we understand the square root of its scalar product with itself, i.e. $\|X\| = \sqrt{X \cdot X}$. Technically, minimisation of Q was implemented using the built-in MATLAB function 'fminsearch' which uses the Nelder-Mead simplex algorithm procedure of finding the minimum of a multivariable function. The detailed description of the Nelder-Mead algorithm can be found in the textbooks on optimisation[2].

The brief pseudo-code showing how the function Q can be computed numerically is provided below.

Pseudo-code for constructing function Q

(1) Set the matrix P using parameters α_i, β_i, and γ_i (see the above table for P).

(2) Set (initially) Q =0.

(3) Run simulations for l different initial conditions (l=6 starting variants):

for i=1:l

(i) Download the final distributions $Y_m(i)$ from data (i corresponds to any of A_2, B, C, D_2, E, F); m is the number of experimental set for the given i here m=1,2,.. $k(i)$.

(ii) Set the current initial distribution X_0 starting from 100% of bacteria from the given variant i.

(iii) Run the model for n=20 time iterations (cell divisions):

for j=1: n

$X_j = X_{j-1} * P$;

end

For each m find the difference between the model prediction and data:

for m=1: $k(i)$

$W_m = X_n - Y_m(i)$

end

Compute the increment in Q corresponding to variant i:

for m=1: $k(i)$

$Q = Q + W_m * W_m$ (symbol '*' signifies the scalar product)

end

end

(4) Print the final Q.

In this study we explored both cases where α_i, β_i, γ_i were distinct parameter for i=1,2,3 (Model 1) and the case where they are identical (Model 2), i.e. when $\alpha_1 = \alpha_2 = \alpha_3$, $\beta_1 = \beta_2 = \beta_3$; $\gamma_1 = \gamma_2 = \gamma_3$. To compare the two models, we applied the Akaike information criterion by calculating the Akaike weights. The corresponding Akaike weight is calculated as

$$w = \frac{exp((\Delta AIC)/2)}{exp((\Delta AIC)/2) + 1},$$

where ΔAIC is the difference in the Akaike score AIC between the models with the minimal and the maximal scores[3]. For the Akaike score of a model with m parameters and N experiments we can use the following approximation

$$AIC = 2m + Nln(Q_{min}),$$

where Q_{min} is the minimised value of Q.

We obtained the following estimates for the parameters:

for **Model 1**: α_1=0.0066, β_1=0.0006, γ_1=0.0012, α_2=0.0013, β_2=0.0189, γ_2=0.0005, α_2=0.0065, β_2=0.0007, γ_2=0.0019, m=9.

for **Model 2**: α=0.0779, β=0.1063, γ=0.0178, m=3.

Calculation of the Akaike weight gave $w \approx exp(-55)$ <<1, which clearly favours Model 1.

1. Gagniuc, P. A. *Markov Chains: From Theory to Implementation and Experimentation.* (John Wiley & Sons , 2017).
2. Nash, J. C. *Compact Numerical Methods: Linear Algebra and Function Minimisation.* (Adam Hilger Ltd, 1979).
3. Burnham, K. P.; Anderson, D. R. *Model Selection and Multimodel Inference: A practical information-theoretic approach*. (Springer-Verlag, 2002).

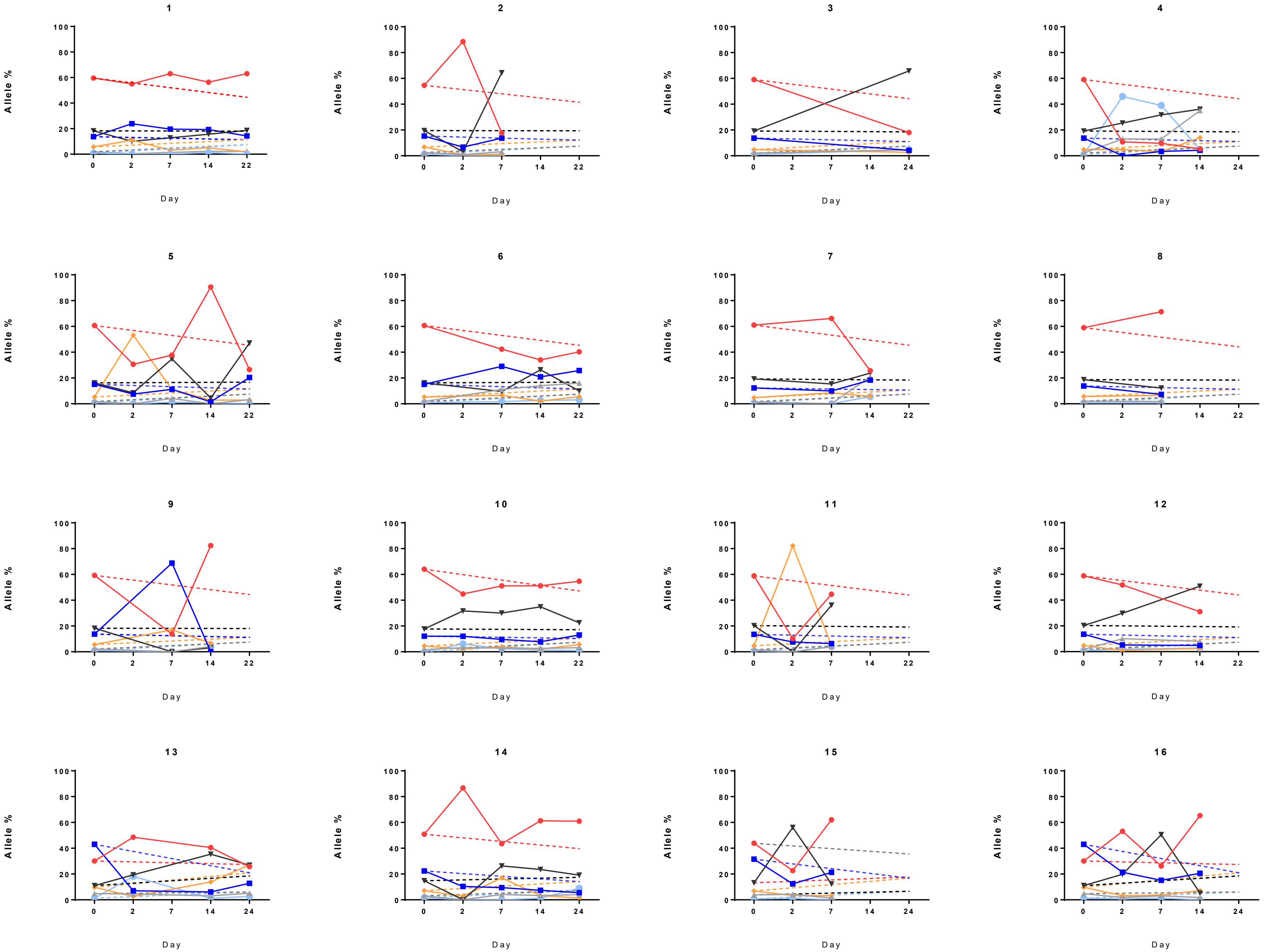
1
2
3
4
5
6
7
8
9
10
11
12
13
14
15
16
Allele %
Day

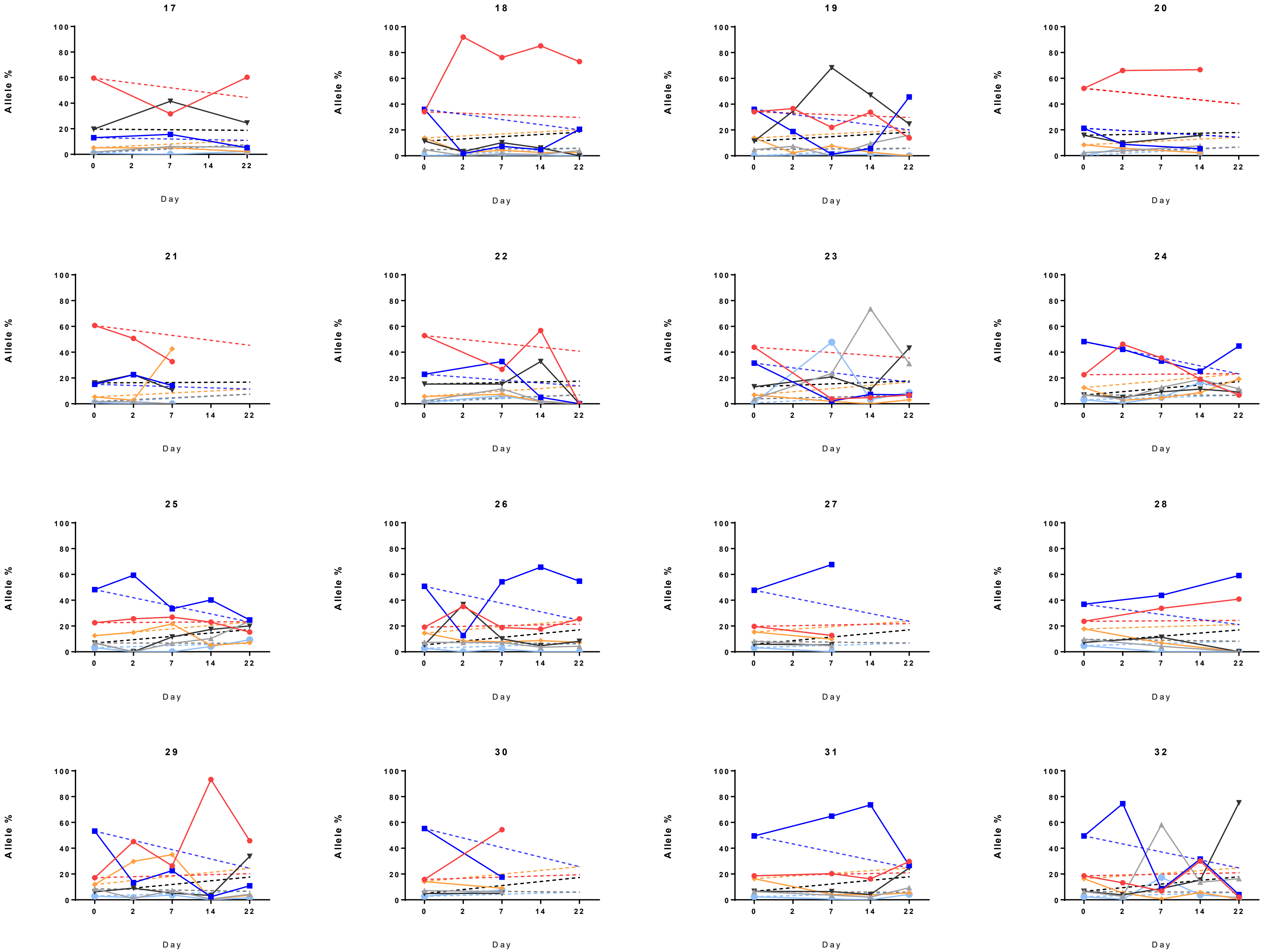
17
18
19
20
21
22
23
24
25
26
27
28
29
30
31
32
Allele %
Day
0
20
40
60
80
100
0
2
7
14
22

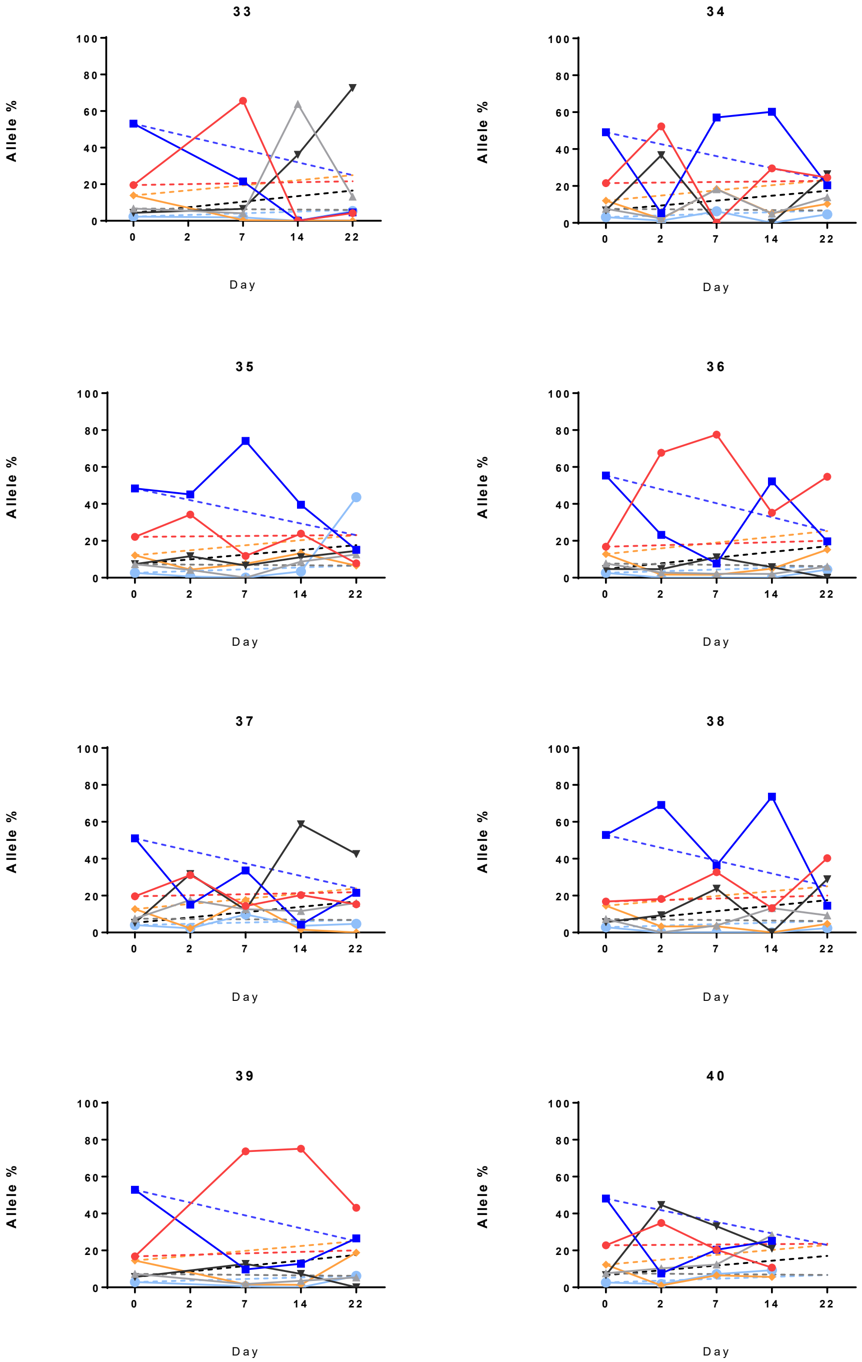
33
34
35
36
37
38
39
40
Allele %
Day
0
2
7
14
22
0
20
40
60
80
100

Figure S1 - The observed and modelled active *hsdS* distribution in pneumococci recovered from the nasopharynx of individual volunteers

The initial active *hsdS* gene distributions within inoculating doses of *S. pneumoniae* BHN418 were experimentally determined. This represents time point zero for both modelled (dashed lines) and experimentally quantified (solid lines) datasets. Experimentally quantified *hsdS* distributions are shown for individual volunteers (one volunteer per graph) over time as *hsdSA* (red), *hsdSB* (dark blue), *hsdSC* (grey), *hsdSD* (black), *hsdSE* (yellow) and *hsdSF (*light blue). Not all volunteers were carriage positive up to day 22, and for some time points fewer than 10 colonies were recovered and therefore not analysed.

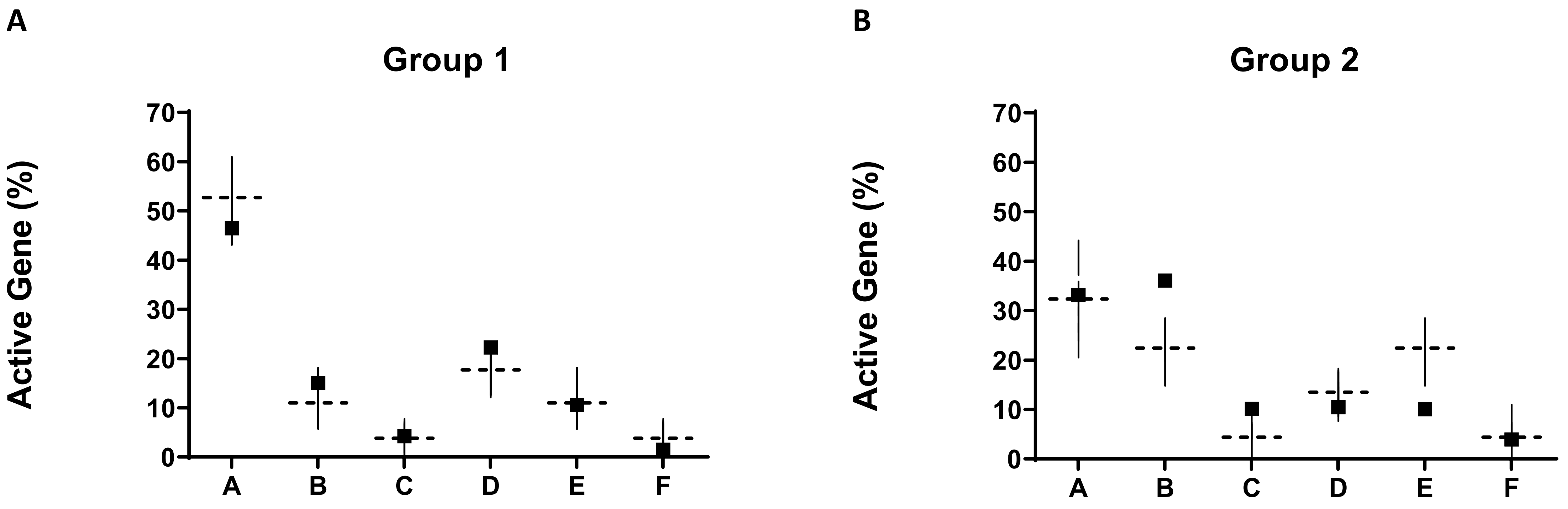

Figure S2- The observed versed expected active *hsdS* distribution in pneumococci recovered from the nasopharynx of individual volunteers at 7 days post colonisation

The initial active *hsdS* gene distributions within inoculating doses of *S. pneumoniae* BHN418 were experimentally determined. Experimentally quantified time points include all volunteers carriage positive by PCR. When the experimental outcomes of group one (panel A) and group two (panel B) were compared to the outcome predicted by the model at 7 days (84 generations) there were no significant differences were observed. Model range is shown by the vertical line, mean percentage of active genes is shown by squares. Significance was tested used a student's T test with a Holm-Sidak correction, *p <0.05, **p =0.01, ***p =0.001, ****p <0.001.
